# An Iterative Nonlinear Filter Using Variational Bayesian Optimization

**DOI:** 10.3390/s18124222

**Published:** 2018-12-01

**Authors:** Yumei Hu, Xuezhi Wang, Hua Lan, Zengfu Wang, Bill Moran, Quan Pan

**Affiliations:** 1 School of Automation, Northwestern Polytechnical University, Xi’an 710072, China; hym@mail.nwpu.edu.cn (Y.H.); wangzengfu@gmail.com (Z.W.); quanpan@nwpu.edu.cn (Q.P.); 2 Key Laboratory of Information Fusion Technology, Ministry of Education, Xi’an 710072, China; 3 School of Engineering, RMIT University, Melbourne 3000, Australia; xuezhi.wang@rmit.edu.au; 4 Department of Electrical and Electronic Engineering, University of Melbourne, Melbourne, VIC 3010, Australia; wmoran@unimelb.edu.au

**Keywords:** target tracking, nonlinear filtering, variational Bayes, Kullback-Leibler divergence

## Abstract

We propose an iterative nonlinear estimator based on the technique of variational Bayesian optimization. The posterior distribution of the underlying system state is approximated by a solvable variational distribution approached iteratively using evidence lower bound optimization subject to a minimal weighted Kullback-Leibler divergence, where a penalty factor is considered to adjust the step size of the iteration. Based on linearization, the iterative nonlinear filter is derived in a closed-form. The performance of the proposed algorithm is compared with several nonlinear filters in the literature using simulated target tracking examples.

## 1. Introduction

Bayesian estimation is widely applied across many areas of engineering such as target tracking, aerial surveillance, intelligent vehicles, and machine learning [1]. In linear Gaussian systems, the state estimation can be optimally achieved using a Kalman filter as a closed form solution. However, many real-world estimation problems are nonlinear, resulting in analytically intractable posterior probability density function (PDF) for the state. In consequence, suboptimal approximation methods are sought to solve the nonlinear estimation problems [2].

Many suboptimal techniques have been developed to solve nonlinear estimation problems. These techniques may be divided into the following three categories. The first category, includes the extended Kalman filter (EKF) [3], the iterated extended Kalman filter (IEKF) [4,5], and their variants [6,7], solves the state estimation problem through replacing the nonlinear functions by their linear approximations via the Taylor expansion. The second category involves stochastic sampling methods. In the filtering process, a set of randomly sampled points with weights are adopted to approximate the PDF of the underlying state. For example, the particle filter (PF) [8,9,10] is a sequential Monte Carlo (SMC) stochastic sampling method, which approximates the PDF by the sampled particles from a proposal distribution. PF can be applied to nonlinear non-Gaussian systems. Markov Chain Monte Carlo (MCMC) is another popular stochastic method since it is able to achieve arbitrarily high accuracy using a large number of particles, sometimes resulting in prohibitive computational expenditure. The techniques in the third category use deterministic sampling methods. Nonlinear state PDFs are approximated by a set of fixed points and weights that represent the location and spread of the distribution. This category includes the unscented Kalman filter (UKF) [9,11,12], the cubature Kalman filter (CKF) [13,14], and the central difference Kalman filter (CDKF) [15]. The UKF and the CDKF approximate the nonlinear state transition function and the measurement function by the unscented transform (UT) and the Stirling interpolation, respectively. The state and the corresponding error covariance are then calculated based on the sampling points and the weights. The CKF uses the third order spherical-radial cubature rule to approximate integrals numerically.

Where nonlinear filters involve optimisation, quasi-Newton approximations are used, along with Kullback-Leibler (KL) divergence and α-divergence as objective functions. For example, the EKF update step may use a Hessian correction term, resulting in improved performance [7,16]. Another approach for estimation, in some ways can be regarded as the fourth category, uses the variational Bayes (VB) technique. VB has a unified and principled architecture and can be used, as with other methods mentioned earlier, for problems where analytic solutions cannot be found. The objective of the VB approximation is to find the variational distribution best able, in the sense of the KL divergence, to approximate the true PDF [17,18,19]. Various variational inference methods for parametric distributions are discussed in [20], where the true PDF of hidden variables is assumed to be given by a parametric model. Since VB usually runs faster than MCMC [20,21,22], it is widely applied in areas such as statistics, finite element analysis, machine learning, etc. An important concern with the VB inference is the accuracy of the approximation [23]. Paul [24] and Stephane et al. [25] introduced a proximal point algorithm in the framework of the expectation-maximization (EM) and studied its convergence. Khan et al. [18,26] adopted KL divergence as a measure of divergence to improve the accuracy of VB inference by considering the geometry of the true posterior PDF. They applied their algorithm to parameter estimation, data set classification and regression.

In this paper, we adopt KL divergence as a metric of posterior PDF approximation, and derive an alternative nonlinear estimation algorithm based on the VB technique. The posterior PDF of the underlying system state is approximated by a parameterized variational distribution and the difference between the two distributions is minimized iteratively using a weighted KL divergence criterion. This is carried out through optimization of the evidence lower bound (ELBO); the intractable integration of the posterior PDF is converted to a solvable optimization problem. A penalty factor is applied to ensure that the filter algorithm obtains a good trade-off between accuracy and computational overhead. Numerical simulations of two typical target tracking scenarios and a benchmark nonlinear filtering problem are presented. The simulation results show that the approximation of nonlinear stochastic system state by the proposed algorithm is tighter than EKF and UKF. The computational cost and the effect of different penalty factors on estimation accuracy are analyzed.

The rest of the paper is organized as follows. In Section 2, we introduce the general nonlinear Bayesian filtering problem. The formulation of the ELBO is described in Section 3. In Section 4, we propose the proximal iterative nonlinear filter. In Section 5, we present simulations of two target tracking scenarios, where the performance of the proposed algorithm is compared with those of EKF and UKF in terms of estimation accuracy and computational overhead. Lastly, conclusions are drawn in Section 6.

## 2. Problem Formulation

Consider a general dynamic system with measurement as follows.

(1) $$x_{k}=f_{k}\left(x_{k-1}\right)+\omega_{k},$$

(2) $$z_{k}=h_{k}\left(x_{k}\right)+v_{k},$$

where $f_{k}\left(\cdot \right)$ denotes the state transition function, and $h_{k}\left(\cdot \right)$ denotes the mapping from the system state to the measurement; $\omega_{k}$ and $v_{k}$ are the process noise and the measurement noise, respectively. We assume that $\omega_{k}$ and $v_{k}$ are Gaussian and mutually independent, $\omega_{k}∼N\left(0,Q_{k}\right)$ and $v_{k}∼N\left(0,R_{k}\right)$.

Assuming that the posterior PDF $p\left(x_{k-1}|z_{k-1}\right)$ at time k−1 is available, the PDF of the predicted state is obtained by the Chapman-Kolmogorov equation.

(3) $$p\left(x_{k}|z_{k-1}\right)=\int p\left(x_{k}|x_{k-1}\right)p\left(x_{k-1}|z_{k-1}\right)dx_{k-1}.$$

Then, at time *k*, the posterior PDF is obtained using the measurement $z_{k}$ by an application of the Bayes rule:

(4) $$p\left(x_{k}|z_{k}\right)=\frac{p\left(z_{k}|x_{k}\right)p\left(x_{k}|z_{k-1}\right)}{\int p\left(z_{k}|x_{k}\right)p\left(x_{k}|z_{k-1}\right)dx_{k}}.$$

For linear Gaussian systems, it is well known that the optimal state estimation $x_{k|k}$ and the corresponding error covariance $P_{k|k}$ under the criterion of minimum variance estimation are obtained by:

(5) $$x_{k|k}=\int x_{k}p\left(x_{k}|z_{k}\right)dx_{k},$$

(6) $$P_{k|k}=\int \left(x_{k}-x_{k|k}\right){\left(x_{k}-x_{k|k}\right)}^{T}p\left(x_{k}|z_{k}\right)dx_{k}.$$

For nonlinear systems, the integral in Equation (4) is often intractable. Suboptimal approximations for the posterior PDF are needed. Most existing suboptimal algorithms adopt linearization or sampling techniques to approximate the posterior PDF $p\left(x_{k}|z_{k}\right)$. We consider an iterative VB approach, in which the true PDF is approximated by a variational distribution and is approached by iterative optimization of the ELBO. The proposed algorithm converts the nontrivial integration to a closed-form optimization and therefore improves estimation accuracy.

## 3. Evidence Lower Bound Maximization

The above nonlinear estimation problem can be solved using a VB framework. Express the marginal PDF $p\left(z_{k}\right)$ using a variational distribution $q\left(x_{k}|\psi_{k}\right)$ as follows:

(7) $$\begin{array}{cc} \log p\left(z_{k}\right) & =\log\int q\left(x_{k}|\psi_{k}\right)\frac{p\left(x_{k},z_{k}\right)}{q\left(x_{k}|\psi_{k}\right)}dx_{k}\\ & =L\left(\psi_{k}\right)+D_{\mathrm{KL}}\left[p\left(x_{k}|z_{k}\right)∥q\left(x_{k}|\psi_{k}\right)\right], \end{array}$$

where $D_{\mathrm{KL}}\left[p\left(x_{k}|z_{k}\right)∥q\left(x_{k}|\psi_{k}\right)\right]$ is the KL divergence between the true posterior PDF $p(x_{k}|z_{k})$ and the variational distribution $q(x_{k}|\psi_{k})$; that is,

(8) $$D_{\mathrm{KL}}\left[p\left(x_{k}|z_{k}\right)∥q\left(x_{k}|\psi_{k}\right)\right]=-\int q\left(x_{k}|\psi_{k}\right)\log\frac{p\left(x_{k}|z_{k}\right)}{q\left(x_{k}|\psi_{k}\right)}dx_{k}.$$

$L\left(\psi_{k}\right)$ is the variational ELBO:

(9) $$\begin{array}{cc} L\left(\psi_{k}\right) & =\int q\left(x_{k}|\psi_{k}\right)\log\frac{p\left(x_{k},z_{k}\right)}{q\left(x_{k}|\psi_{k}\right)}dx_{k}\\ & =E_{q}\left[\log p\left(z_{k}|x_{k}\right)\right]-D_{\mathrm{KL}}\left[p\left(x_{k}\right)∥q\left(x_{k}|\psi_{k}\right)\right]. \end{array}$$

The variational distribution $q\left(x_{k}|\psi_{k}\right)$ is assumed Gaussian with unknown parameter $\psi_{k}=\left(x_{k|k},\;P_{k|k}\right)$ (to be estimated), where $x_{k|k}$ is the mean and $P_{k|k}$ is the covariance.

Please note that the poterior PDF $p(x_{k}|z_{k})$ needs to be closely approximated by a known distribution in nonlinear filtering. From Equation (7), evidently, the variational distribution $q(x_{k}|\phi_{k})$ would be equal to the true posterior PDF $p(x_{k}|z_{k})$ if the KL divergence were zero. Minimizing the KL divergence, and thereby approximating the posterior PDF by a variational distribution, is equivalent to maximizing the ELBO, i.e.,

(10) $$\begin{array}{cc} \psi_{k}^{*} & =\underset{\psi_{k}}{\arg\max}\;L\left(\psi_{k}\right). \end{array}$$

According to Equations (9) and (10), the nontrivial integration of Equation (7) is converted to the problem of maximizing ELBO, which can be solved by the VB method.

## 4. Proximal Iterative Nonlinear Filter

In this section, we derive an iterative procedure in a closed-form to iteratively maximize the ELBO so as to minimize the KL divergence between the true posterior PDF $p(x_{k}|z_{k})$ and the variational distribution $q(x_{k}|\psi_{k})$.

### 4.1. Penalty Function Based on KL Divergence

Notice that the KL divergence $D_{\mathrm{KL}}\left[q\left(x_{k}|\psi_{k}\right)∥q\left(x_{k}|\psi_{k}^{i}\right)\right]$ is nonnegative for all $q\left(x_{k}|\psi_{k}\right)$. Following [24], we adopt the proximal point algorithm to generate a sequence $\psi_{k}^{i+1}$ via the following iterative scheme,

(11) $$\psi_{k}^{i+1}=\underset{\psi_{k}}{\arg\max}L\left(\psi_{k}\right)-\frac{1}{\beta_{i}}D_{\mathrm{KL}}\left[q\left(x_{k}|\psi_{k}\right)∥q\left(x_{k}|\psi_{k}^{i}\right)\right],$$

where *i* denotes the iteration index and the penalty factor $\beta_{i}$ is used to adjust the optimization step length. Roughly speaking, the ELBO is maximized when the KL divergence between the two variational distributions $q\left(x_{k}|\psi_{k}\right)$ and $q\left(x_{k}|\psi_{k}^{i}\right)$ approaches zero. Equation (11) can be rewritten as

(12) $$\psi_{k}^{i+1}=\underset{\psi_{k}}{\arg\max}\left\{L\left(\psi_{k}\right)-\frac{1}{\beta_{i}}D_{\mathrm{KL}}\left[q\left(x_{k}|\psi_{k}\right)∥q\left(x_{k}|\psi_{k}^{i}\right)\right]\right\}.$$

Please note that one iteration of this proximal method is equivalent to moving a step in the direction of the natural gradient [18]. The influence of the KL divergence on $\psi_{k}^{i+1}$ can be adjusted by $\beta_{i}$. The larger the $\beta_{i}$, the weaker the influence of the KL divergence on $\psi_{k}^{i+1}$, and vice versa. In [18], it is assumed that $\beta_{i}=1$.

### 4.2. The Proximal Iterative Nonlinear Filter

The proximal iterative method is implemented via an iterative minimization of the KL divergence, where the initial state is assigned with an estimation from a core-filter, e.g., Bayesian filter. Here, EKF is adopted as the core-filter to predict and update the system state before the iterative optimization process. We propose a proximal iterative nonlinear filter combined with VB, called PEKF-VB, which is described and derived in the following.

Firstly, by substituting Equation (10) into Equation (12), we can rewrite the iterative optimization as

(13) $$\begin{array}{c} \psi_{k}^{i+1}\underset{\psi_{k}}{=\arg\max}\left\{E_{q}\left[\log p\left(z_{k}|x_{k}\right)\right]-D_{\mathrm{KL}}\left[p\left(x_{k}\right)∥q\left(x_{k}|\psi_{k}\right)\right]-\frac{1}{\beta_{i}}D_{\mathrm{KL}}\left[q\left(x_{k}|\psi_{k}\right)∥q\left(x_{k}|\psi_{k}^{i}\right)\right]\right\}. \end{array}$$

Under the Gaussian assumptions for the process noise and the measurement noise, the variational distribution is of the form $q\left(x_{k}|\psi_{k}\right)∼N\left(x_{k};\;x_{k|k},P_{k|k}\right)$. Given the prior of the state $x_{k-1|k-1}$ at time k−1, we can assume $p\left(x_{k}\right)∼N\left(x_{k};\;x_{k|k-1},P_{k|k-1}\right)$. Accordingly, the $\psi_{k}^{i+1}$ is

(14) $$\begin{array}{cc} \psi_{k}^{i+1}= & \;\underset{\psi_{k}}{\arg\max}\left\{E_{q}\left[\log p\left(z_{k}|x_{k}\right)\right]-D_{\mathrm{KL}}\left[N\left(x_{k};\;x_{k|k-1},P_{k|k-1}\right)∥N\left(x_{k};\;x_{k|k},P_{k|k}\right)\right]\right.\\ & \left.-\frac{1}{\beta_{i}}D_{\mathrm{KL}}\left[q\left(x_{k}|\psi_{k}\right)∥q\left(x_{k}|\psi_{k}^{i}\right)\right]\right\}. \end{array}$$

For the first term in Equation (14), the expectation $E_{q}\left[\log p\left(z_{k}|x_{k}\right)\right]$ related to $x_{k}$ and $P_{k}$ can be approximated linearly using the gradients with respect to (w. r. t.) $x_{k}$ and $P_{k}$. Defining $g\left(x_{k|k},P_{k|k}\right)≜E_{q}\left[\log p\left(z_{k}|x_{k}\right)\right]$, the gradients of *g* w. r. t. $x_{k|k}$ and $P_{k|k}$ are

(15) $$\nabla_{x}g\left(x_{k|k},P_{k|k}\right)=-\alpha_{k},$$

(16) $$\nabla_{P}g\left(x_{k|k},P_{k|k}\right)=-\gamma_{k}.$$

The expectation $E_{q}\left[\log p\left(z_{k}|x_{k}\right)\right]$ at time *k* is then maximized by gradient ascent in the variables $x_{k|k}$ and $P_{k|k}$; that is,

(17) $$\begin{array}{c} E_{q}\left[\log p\left(z_{k}|x_{k}\right)\right]\approx \alpha_{k}x_{k|k}+\frac{1}{2}\gamma_{k}P_{k|k}, \end{array}$$

where $\alpha_{k}^{i}$ and $\gamma_{k}^{i}$ at iteration *i* are given by

(18) $$\alpha_{k}^{i}=-{\left(H_{k}^{i}\right)}^{T}R_{k}^{-1}\left(z_{k}\;-h_{k}\left(x_{k|k}^{i}\right)\right),$$

(19) $$\gamma_{k}^{i}=\frac{1}{2}{\left(H_{k}^{i}\right)}^{T}R_{k}^{-1}H_{k}^{i},$$

and $H_{k}^{i}$ is the Jacobian matrix:

(20) $$H_{k}^{i}=\frac{\partial h_{k}\left(x\right)}{\partial x^{T}}{|}_{x=x_{k|k}^{i}}.$$

In other words, the coefficients $\alpha_{k}^{i}$ and $\gamma_{k}^{i}$ are updated by $H_{k}^{i}$ with $x_{k|k}^{i}$ in each iterative step. The detailed calculations of $\alpha_{k}^{i}$ and $\gamma_{k}^{i}$ are given in Appendix A.

For Gaussian distributions $N\left(\xi_{1};\;\mu_{1},C_{1}\right)$ and $N\left(\xi_{2};\;\mu_{2},C_{2}\right)$ with the same dimension *d*, the KL divergence is

(21) $$\begin{array}{c} D_{\mathrm{KL}}\left[N\left(\xi_{1};\;\mu_{1},C_{1}\right)∥N\left(\xi_{2};\;\mu_{2},C_{2}\right)\right]=\;-\frac{1}{2}\left[\log\left|C_{1}C_{2}^{-1}\right|-\;\mathrm{tr}\left(C_{1}C_{2}^{-1}\right)-\;{\left(\mu_{1}-\mu_{2}\right)}^{T}C_{2}^{-1}\left(\mu_{1}-\mu_{2}\right)+d\right]. \end{array}$$

where operators tr(·) and $\left|\cdot \right|$ denote the trace and the determinant of a matrix, respectively. By Equations (17)–(21), Equation (13) can be rewritten as,

(22) $$\begin{array}{cc} \psi_{k}^{i+1}= & \underset{\psi_{k}}{\arg\max}\left\{\alpha_{k}^{i}x_{k|k}+\frac{1}{2}\gamma_{k}^{i}P_{k|k}-\frac{1}{2}\left[\log\left|P_{k|k-1}{\left(P_{k|k}^{i}\right)}^{-1}\right|-\mathrm{tr}\left(P_{k|k-1}P_{k|k}^{-1}\right)\right.\right.\\ & \left.\left.-\mathrm{tr}\left(P_{k|k}{\left(P_{k|k}^{i}\right)}^{-1}\right)-{\left(x_{k|k-1}-x_{k|k}\right)}^{T}P_{k|k}^{-1}\left(x_{k|k-1}-x_{k|k}\right)\right.\right.\\ & \left.\left.-{\left(x_{k|k}-x_{k|k}^{i}\right)}^{T}{\left(P_{k|k}^{i}\right)}^{-1}\left(x_{k|k}-x_{k|k}^{i}\right)\right]-d\right\}. \end{array}$$

Then $\psi_{k}^{i+1}$ is maximized at a point (x,P) which can be explicitly calculated. To find them, set the partial derivatives of $\psi_{k}^{i+1}$ w.r.t. {x,P} to be zero, i.e.,

(23) $$\frac{\partial \psi_{k}^{i+1}}{\partial x}{{|}_{x=x_{k|k}^{i+1}}=0,\;\;\frac{\partial \psi_{k}^{i+1}}{\partial P}|}_{P=P_{k|k}^{i+1}}=0.$$

**Figure 1:**
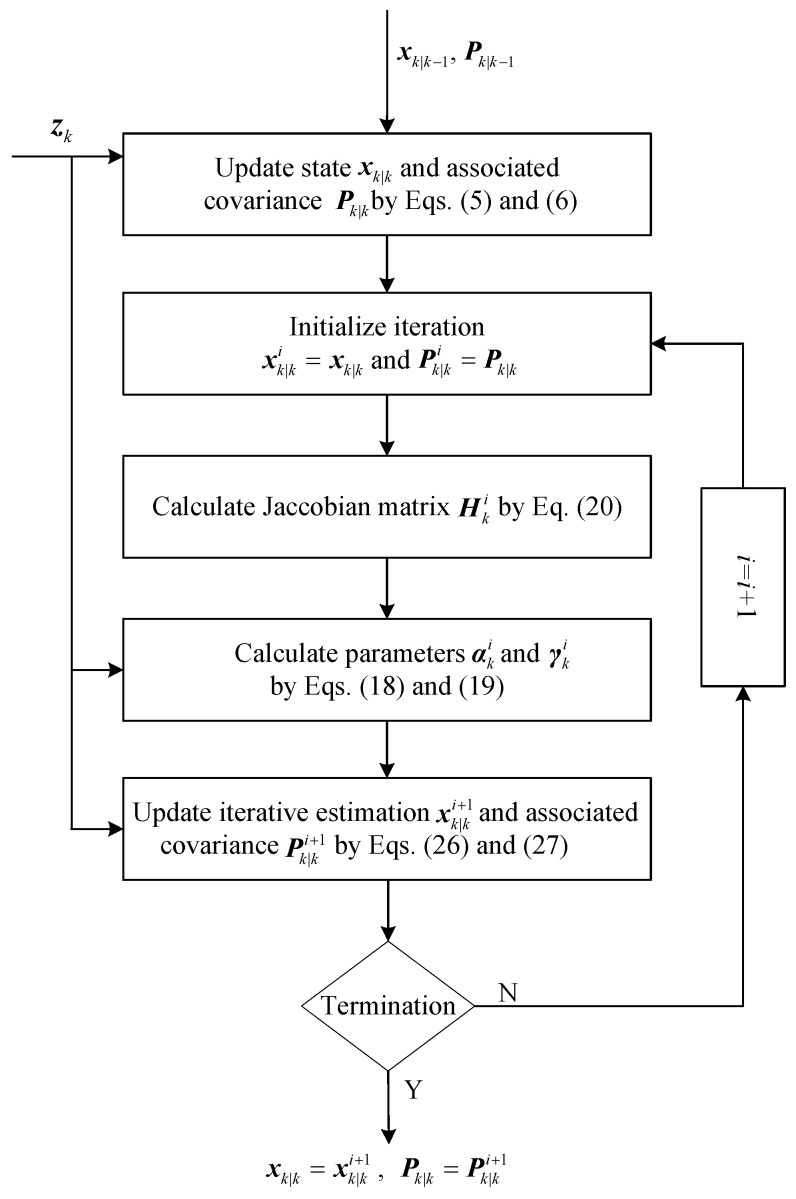
The flow diagram of the proposed PEKF-VB algorithm.

Accordingly, $x_{k|k}^{i+1}$ and $p_{k|k}^{i+1}$ are seen to be

(24) $$\begin{array}{cc} x_{k|k}^{i+1} & ={\left[\frac{\beta_{i}}{1+\beta_{i}}P_{k|k-1}^{-1}+\frac{1}{1+\beta_{i}}{\left(P_{k|k}^{i}\right)}^{-1}\right]}^{-1}\left[\frac{\beta_{i}}{1+\beta_{i}}\left(P_{k|k-1}^{-1}x_{k|k-1}-\alpha_{k}^{i}\right)+\frac{1}{1+\beta_{i}}{\left(P_{k|k}^{i}\right)}^{-1}x_{k|k}^{i}\right]\\ & ={\left[\left(1-b_{i}\right)P_{k|k-1}^{-1}+b_{i}{\left(P_{k|k}^{i}\right)}^{-1}\right]}^{-1}\left[\left(1-b_{i}\right)\left(P_{k|k-1}^{-1}x_{k|k-1}-\alpha_{k}^{i}\right)+b_{i}{\left(P_{k|k}^{i}\right)}^{-1}x_{k|k}^{i}\right], \end{array}$$

(25) $$\begin{array}{cc} P_{k|k}^{i+1} & ={\left[\frac{1}{1+\beta_{i}}{\left(P_{k|k}^{i}\right)}^{-1}\;\;\;+\frac{\beta_{i}}{1+\beta_{i}}\left(P_{k|k-1}^{-1}+\gamma_{k}^{i}\right)\right]}^{-1}\;\;\;={\left[b_{i}{\left(P_{k|k}^{i}\right)}^{-1}\;\;\;\;\;+\left(1-b_{i}\right)\left(P_{k|k-1}^{-1}+\gamma_{k}^{i}\right)\right]}^{-1}, \end{array}$$

where $b_{i}=\frac{1}{1+\beta_{i}}$. Equations (24) and (25) show that the state estimate and the associated covariance in the iteration are updated by $\alpha_{k}^{i}$ and $\gamma_{k}^{i}$, respectively. As shown in Figure 1, the complete iteration procedure consists of Equations (18), (19), (24) and (25).

We note that, in principle, the computational cost in Equations (24) and (25) can be slightly reduced by using the Matrix Inversion Lemma [27]. As a result, Equations (24) and (25) are derived as Equations (26) and (27), respectively.

(26) $$x_{k|k}^{i+1}=\left\{\frac{1}{b_{i}}P_{k|k}^{i}\;\;-\frac{1}{b_{i}}P_{k|k}^{i}\left[\frac{1}{1-b_{i}}P_{k|k-1}\;\;+\frac{1}{b_{i}}P_{k|k}^{i}\right]\frac{1}{b_{i}}P_{k|k}^{i}\right\}\left[\left(1-b_{i}\right)\left(P_{k|k-1}^{-1}x_{k|k-1}-\alpha_{k}^{i}\right)+b_{i}{\left(P_{k|k}^{i}\right)}^{-1}\;\;x_{k|k}^{i}\right]$$

(27) $$P_{k|k}^{i+1}=\frac{1}{1-b_{i}}K_{k}^{-1}-\frac{b_{i}}{1-b_{i}}K_{k}^{-1}{\left(P_{k|k}^{i}\right)}^{-1}{\left(I_{4\times 4}+\frac{b_{i}}{1-b_{i}}K_{k}^{-1}{\left(P_{k|k}^{i}\right)}^{-1}\right)}^{-1}\frac{1}{1-b_{i}}K_{k}^{-1},$$

where $K_{k}^{-1}={\left(P_{k|k-1}^{-1}+\gamma_{k}^{i}\right)}^{-1}=P_{k|k-1}^{-1}-P_{k|k-1}^{-1}\gamma_{k}^{i}\left(I_{4\times 4}+P_{k|k-1}\gamma_{k}^{i}\right)P_{k|k-1}$.

For special case when $\beta_{i}=1$, Equations (24) and (25) can be rewritten as

(28) $$\begin{array}{cc} x_{k|k}^{i+1} & =c_{1}x_{k|k-1}+c_{2}x_{k|k}^{i}-c_{g}\alpha_{k}^{i}, \end{array}$$

(29) $$\begin{array}{cc} P_{k|k}^{i+1} & =2{\left(c_{g}^{-1}+\gamma_{k}^{i}\right)}^{-1}, \end{array}$$

where $c_{g}={\left(P_{k|k-1}^{-1}+{\left(P_{k|k}^{i}\right)}^{-1}\right)}^{-1}$, $c_{1}=c_{g}P_{k|k-1}^{-1}$ and $c_{2}=c_{g}{\left(P_{k|k-1}^{i}\right)}^{-1}$.

The flow diagram of the proposed PEKF-VB algorithm is shown in Figure 1 and the detailed implementation of PEKF-VB is given in Algorithm 1.

**Algorithm 1** The implementation of the PEKF-VB algorithm
1:Initialization (k=0): state estimation $x_{0}$ and associated error covariance $P_{0}$, the number of iterations.2:Compute the predicted state $x_{k|k-1}$ and the associated error covariance $P_{k|k-1}$
$$\begin{array}{c} x_{k|k-1}=f_{k}\left(x_{k-1|k-1}\right),\;\;P_{k|k-1}=F_{k}P_{k-1|k-1}F_{k}^{T}+Q_{k-1}, \end{array}$$
where $F_{k}=\frac{\partial f_{k}\left(x\right)}{\partial x}{|}_{x=x_{k-1|k-1}}$.3:Compute the filtering grain $K_{k}$
$$S_{k}=H_{k}P_{k|k-1}H_{k}^{T}+R_{k},\;\;K_{k}=P_{k|k-1}H_{k}^{T}{\left(S_{k}\right)}^{-1},$$
where $H_{k}=\frac{\partial h_{k}\left(x\right)}{\partial x}{|}_{x=x_{k|k-1}}$.4:Update the state estimation $x_{k|k}$ and the associated error covariance $P_{k|k}$
$$\begin{array}{c} x_{k|k}^{*}=x_{k|k-1}-K_{k}\left(z_{k}-h_{k}\left(x_{k|k-1}\right)\right),\;\;P_{k|k}^{*}=P_{k|k-1}-K_{k}H_{k}P_{k|k-1}. \end{array}$$5:Let $x_{k|k}^{1}=x_{k|k}^{*}$, $P_{k|k}^{1}=P_{k|k}^{*}$, and i=1.6:**while** not converge **do**7: Compute parameters $\alpha_{k}^{i+1}$ and $\gamma_{k}^{i+1}$ by Equations (18) and (19).8: Compute iterated state estimation $x_{k|k}^{i+1}$ and its error covariance $P_{k|k}^{i+1}$ by Equations (26) and (27).9: Let i=i+1.10:
**end while**
11:Let k=k+1, go back to Step 2.


### 4.3. Remarks

1. The VB method approximates the true posterior PDF by choosing from a parameterized variational distribution. In each iteration of the PEKF-VB, the ELBO (9) increases. It follows that the ELBO is a proper criterion for measuring the performance of variational optimization. The ELBO of the proposed nonlinear filter is
  (30) $$\begin{array}{cc} L\left(\psi_{k}\right)= & -\frac{1}{2}\log\frac{{\left(2\pi \right)}^{D_{z}}|R_{k}\left|\right|P_{k|k-1}|}{|P_{k|k}|}-\frac{1}{2}\mathrm{tr}\left(P_{k|k-1}^{-1}\left[\left(x_{k|k}-x_{k|k-1}\right){\left(x_{k|k}-x_{k|k-1}\right)}^{T}\right]\right)\\ & -\frac{1}{2}\mathrm{tr}\left(P_{k|k-1}^{-1}P_{k|k}\right)-\frac{1}{2}\mathrm{tr}\left(R_{k}^{-1}\left(\left(z_{k}-H_{k}x_{k|k}\right){\left(z_{k}-H_{k}x_{k|k}\right)}^{T}+H_{k}P_{k|k}H_{k}^{T}\right)\right)+\frac{D_{x}}{2}, \end{array}$$
  where $D_{x}$ and $D_{z}$ denote the dimension of the state and the dimension of the measurement, respectively. The derivation of the ELBO is given in Appendix B.
2. Apart from the KL divergence, we can use Calvo and Oller’s distance (COD) as the penalty function in Equation (13); the corresponding filter is denoted by CODEKF. The COD of two distributions $f\left(\xi_{1}\right)=p\left(\xi_{1}|\;\mu_{1},C_{1}\right)$ and $f\left(\xi_{2}\right)=p\left(\xi_{2}|\;\mu_{2},C_{2}\right)$ is [28],
  (31) $$d\left(\xi_{1},\xi_{2}\right)={\left[1/2\sum_{i=1}^{n+1}\ln^{2}\lambda_{i}\right]}^{1/2},$$
  where *n* is the dimension of $\xi_{i}$, i∈{1,2}, $\lambda_{i}$ are the eigenvalues of $f(\Delta \bar{\mu },D)$ with $\bar{\mu }=T^{T}C_{1}^{-1/2}\Delta \mu$, $D=T^{T}C_{1}^{-1/2}C_{2}C_{1}^{-1/2}T$ which is diagonal, and $\Delta \mu =\mu_{2}-\mu_{1}$, $TT^{T}=I$. We replace the KLD in Equation (13) with Equation (31) as follows.
  (32) $$\begin{array}{cc} \psi_{k}^{i+1}= & \underset{\psi_{k}}{\arg\max}\left\{\left(\alpha_{k}^{i}x_{k|k}+\frac{1}{2}\gamma_{k}^{i}P_{k|k}\right)-D_{\mathrm{KL}}\left[p\left(x_{k}\right)∥q\left(x_{k}|\psi_{k}\right)\right]-\frac{1}{\beta_{i}}d\left(\xi_{1},\xi_{2}\right)\right\}. \end{array}$$
3. Since both PEKF-VB and CODEKF involve iterations within the VB framework to minimize the divergence between the posterior PDF and variational distribution, their complexity is increased by the calculation of the Jacobian in each iteration.
4. In PEKF-VB, we use KL divergence to measure the similarity between two distributions. Under Gaussian assumptions for the distributions, a closed-form solution of the variational distribution has been derived. However, the VB framework with the KL divergence can also apply to non-Gaussian distributions. If no closed-form exists, a Monte Carlo method can be used to approximate the divergence. Other measures of dissimilarity between probability distributions, such as the alpha-divergence, the Rényi-divergence and the alpha-beta divergence, can also be used in the VB framework. See [29] and references therein. Unfortunately, in general, no computationally tractable form of the variational distribution can be derived and a Monte Carlo method has to be employed.

## 5. Numerical Simulations

In this section, we present two nonlinear estimation examples of 2D target tracking and a benchmark nonlinear filtering problem to illustrate the performance of PEKF-VB and CODEKF. We compare them with EKF and UKF. The performance is measured by root-mean-squared error (RMSE) of the estimates and the computational overhead.

**Figure 2:**
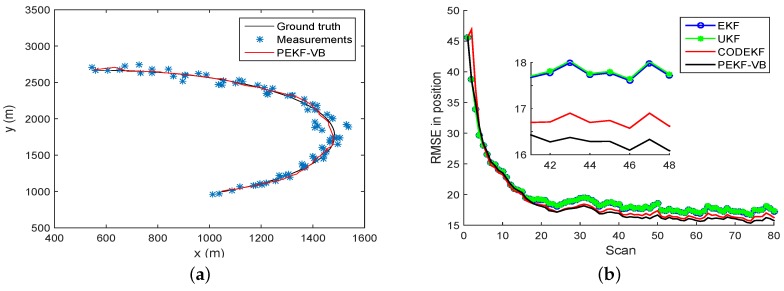
Tracking with a CT model (**a**) Trajectory, measurements and estimation (**b**) RMSE obtained by EKF, UKF, CODEKF and PEKF-VB.

Example 1: Range-bearing tracking. In this scenario, the underlying target motion is described by a constant turn (CT) model, with the state vector consists of 2D position and velocity components. As shown in Figure 2a, the target moves with initial state $x_{0}={\left(565.7,29.99,1166,-0.62\right)}^{T}$ along a circular trajectory, and is observed by a range-bearing sensor. The state transition matrix $F_{k}$ in Equation (1) and the measurement function $h_{k}\left(x_{k}\right)$ in Equation (2) are:

(33) $$F_{k}=\left[\begin{array}{cccc} 1 & \sin\left(\dot{\theta }T\right)/\dot{\theta } & 0 & \left(\cos\left(\dot{\theta }T\right)-1\right)/\dot{\theta }\\ 0 & \cos\left(\dot{\theta }T\right) & 0 & -\sin\left(\dot{\theta }T\right)\\ 0 & \left(1-\cos\left(\dot{\theta }T\right)\right)/\dot{\theta } & 1 & \sin\left(\dot{\theta }T\right)/\dot{\theta }\\ 0 & \sin\left(v\dot{\theta }T\right) & 0 & \cos\left(\dot{\theta }T\right) \end{array}\right],$$

(34) $$h_{k|k}\left(x_{k}\right)=\left[\begin{array}{c} \sqrt{\left(x^{2}+y^{2}\right)}\\ \mathrm{artan}\left(y/x\right) \end{array}\right],$$

where the turn rate $\dot{\theta }=-0.0333\;\mathrm{rad}/s$, the covariances of the zero mean Gaussian white noises $\omega_{k}$ and $\upsilon_{k}$ are $Q_{k}=1.5\;GqG^{T}$ and $R_{k}=\mathrm{diag}\left(r^{2},\;\phi^{2}\right)$, respectively, where $q=I_{2\times 2}$, and $G={\left[\begin{array}{cccc} T^{2}/2 & T & 0 & 0\\ 0 & 0 & T^{2}/2 & T \end{array}\right]}^{T}$, r=35, and ϕ=0.5π/180. At each run, the track is initialised using the two-point method [1] with initial error covariance $P_{0}=\mathrm{diag}\left(\left[600,100,600,100\right]\right)$. We let $\beta_{i}=1$, and T=1s. The target moves for 80 scans (periods). 1000 Monte Carlo runs are carried out.

**Figure 3:**
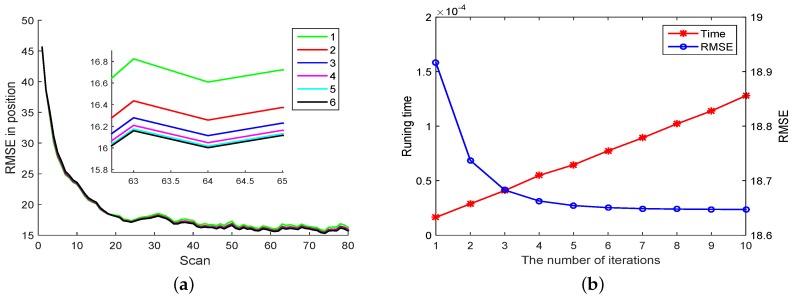
RMSE and running time of PEKF-VB (**a**) RMSE with different number of iterations (**b**) The relationship between iteration and mean RMSE, running time.

The RMSE plots of EKF, UKF, CODEKF and PEKF-VB are showed in Figure 2b. The black, red, green and blue curves are obtained by the PEKF-VB CODEKF, UKF and EKF, respectively. It is seen that in terms of RMSE performance, PEKF-VB is slightly better than CODEF; both PEKF-VB and CODEF are better than EKF and UKF. Table 1 provides the quantitative comparison of RMSE and execution time of EKE, UKF and CODEKF, PEKF-VB. Figure 3b gives the relationship between iteration index and the running time of PEKF-VB.

In Figure 3a we compare the RMSE performance of PEKF-VB by varying the numbers of iterations. Notice that the RMSE decreases with the increasing of the number of iterations. Figure 3b gives the computational overhead of PEKF-VB w.r.t the number of iterations. Both results agree with our intuition.

**Figure 4:**
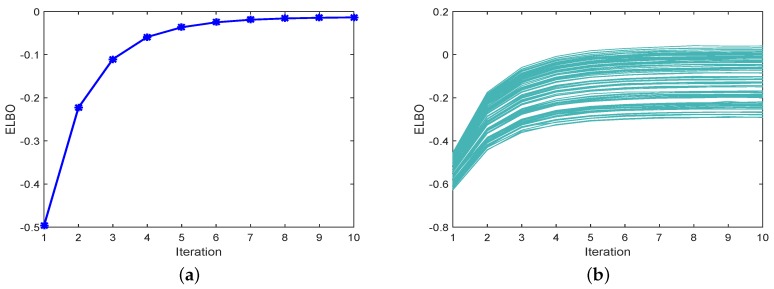
The curve in (**a**) is the ELBO of the second scan. The curves in (**b**) are the ELBO of all scans.

To illustrate the convergence of PEKF-VB, we present the ELBO for different numbers of iterations in Figure 4. Figure 4a illustrates the ELBO at the second scan, and Figure 4b shows the ELBO for all scans. Clearly, the ELBO increases with the number of iterations increases, showing that the iteration procedure in PEKF-VB converges.

**Figure 5:**
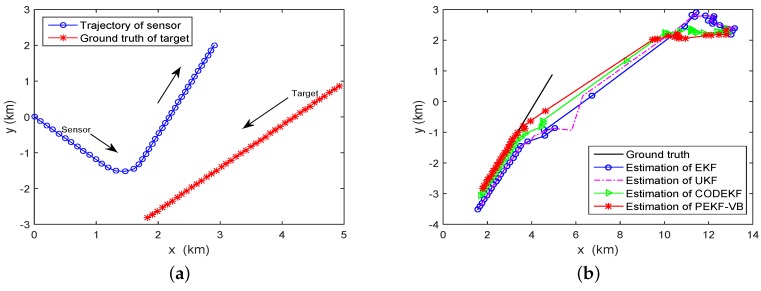
Bearing-only tracking. (**a**) Tracking scenario (**b**) Estimation obtained by EKF, UKF, CODEKF and PEKF-VB.

Example 2: Bearing-only tracking. In this scenario, a single target tracking using measurements from a single bearing-only sensor is considered. While the sensor (ownership) measurement model is nonlinear to the target state, the sensor has to maneuver relative to the target in order to observe it [30,31]. Let $x_{k}=\left[x_{k},y_{k},{\dot{x}}_{k},{\dot{y}}_{k}\right]$ be the state of the target at time *k*, where $\left(x_{k},y_{k}\right)$ and $\left({\dot{x}}_{k},{\dot{y}}_{k}\right)$ are the position and velocity, respectively. The target, with an initial range of 5 km (relative to the sensor) and initial course of 220° in a clockwise direction (Set the positive axis of *y* is 0°), is modeled by a constant velocity model in Equation (35).

(35) $$F_{k}=\left[\begin{array}{cccc} 1 & 0 & T & 0\\ 0 & 1 & 0 & T\\ 0 & 0 & 1 & 0\\ 0 & 0 & 0 & 1 \end{array}\right],$$

where T=1min. The speed of the target is 0.1235 km/min. The sensor starts moving with a fixed speed of 0.1543 km/min and an initial course of 140° in a clockwise direction (Set the positive axis of *y* is 0°). Please note that for the bearing-only tracking problem, to be able to estimate the range of the target, the sensor has to maneuver. Here we assume the sensor maneuvers from scan 14 to scan 17, and then moves with constant velocity from scan 18 to scan 40. The motion model of the sensor is given by Equation (36), where the turn rate $\dot{\theta }=30{}^{\circ}/\min$. Both the target and the sensor move for 40 scans and their trajectories are shown in Figure 5a.

(36) $$\left\{\begin{array}{cc} S_{k}=\left[\begin{array}{cccc} 1 & 0 & T & 0\\ 0 & 1 & 0 & T\\ 0 & 0 & 1 & 0\\ 0 & 0 & 0 & 1 \end{array}\right], & 0<k<14,\;18\leq k\leq 40,\\ S_{k}=\left[\begin{array}{cccc} 1 & 0 & \sin\left(\dot{\theta }T\right)/\dot{\theta } & \left(\cos\left(\dot{\theta }T\right)-1\right)/\dot{\theta }\\ 0 & 1 & \left(1-\cos\left(\dot{\theta }T\right)\right)/\dot{\theta } & \sin\left(\dot{\theta }T\right)/\dot{\theta }\\ 0 & 0 & \cos\left(\dot{\theta }T\right) & -\sin\left(\dot{\theta }T\right)\\ 0 & 0 & \sin\left(\dot{\theta }T\right) & \cos\left(\dot{\theta }T\right) \end{array}\right], & 14\leq k<18. \end{array}\right.$$

The measurement function $h_{k}\left(x_{k}\right)$ of the bearing-only sensor is,

(37) $$h_{k}\left(x_{k}\right)=\mathrm{atan}\left(\left(x_{k}^{\prime }-x_{s}\right)/\left(y_{k}^{\prime }-y_{s}\right)\right),$$

where $x_{k}^{\prime }$ and $y_{k}^{\prime }$ are the position of the target in Cartesian coordinate system, $x_{s}$ and $y_{s}$ are the position of the sensor. The standard deviation of measurement noise is 1°. The initial position of the target is randomly sampled at range r=13km with covariance $P_{0}$

(38) $$P_{0}=ABA^{\prime },$$

where

(39) $$A=\left[\begin{array}{cccc} \cos\dot{\theta } & \sin\dot{\theta } & 0 & 0\\ -\sin\dot{\theta } & \cos\dot{\theta } & 0 & 0\\ 0 & 0 & 1 & 0\\ 0 & 0 & 0 & 1 \end{array}\right],\;B=\left[\begin{array}{cccc} r^{2}\sigma_{m}^{2} & 0 & 0 & 0\\ 0 & \Delta r^{2} & 0 & o\\ 0 & 0 & \Delta v^{2} & 0\\ 0 & 0 & 0 & \Delta v^{2} \end{array}\right].$$

where $\sigma_{m}=\pi /180\;\mathrm{rad}$, Δr=2km and Δv=61.7m/min. We let $\beta_{i}=1$. Figure 5b shows the estimated target trajectories obtained by the EKF, UKF, CODEKF and PEKF-VB for a single run.

**Figure 6:**
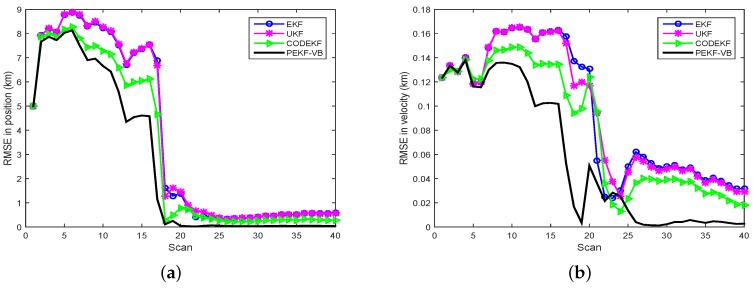
RMSE comparison of EKF, UKF, CODEKF and PEKF-VB in bearing-only tracking. (**a**) RMSE in position (**b**) RMSE in velocity.

Based on 1000 Monte Carlo simulations, the RMSE comparison of EKF, UKF, CODEKF and PEKF-VB is illustrated in Figure 6, where the number of iterations for PEKF-VB is 5.

- As we expected, with a fixed sensor trajectory, PEKF-VB has the best target observability from sensor measurements and leads to a better RMSE performance than EKF and UKF. Under the VB framework, the variational distribution approaches the real posterior PDF through the iteration of the proximal filter.
- The RMSE performance of CODEKF is also better than those of EKF and UKF because, for CODEKF, the Jacobian matrix of Equation (37) in each iteration is updated to minimize the COD. However, the RMSE performance of CODEKF is slightly worse than that of PEKF-VB.
- In the first few scans, the performance of the four filters are comparable. This is because, in this bearing-only tracking problem, the accumulative measurements in these scans do not provide enough information to the four filters. The performance of CODEKF and of PEKF-VB suffers when measurement data is very limited. As more measurements accumulate both CODEKF and PEFK-VB extract more information via the iteration process, resulting in superior performance.

**Figure 7:**
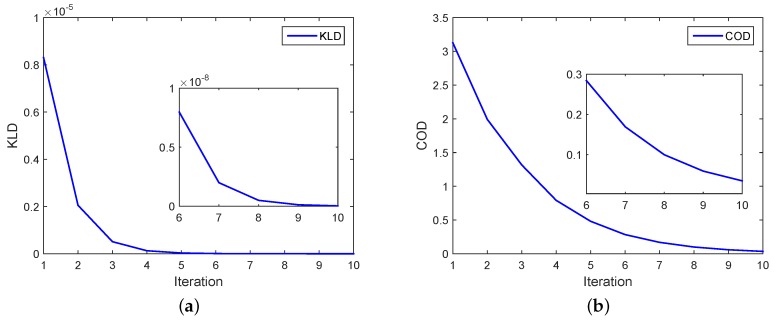
Value of metrics versus the number of iterations for PEKF-VB and CODEKF. (**a**) KL divergence in PEKF-VB, (**b**) COD in CODEKF.

Figure 7 shows the metric values versus the number of iterations for PEKF-VB and CODEKF. The KLD curve is close to zero after the fifth iteration. The enlarged plots marked from the sixth to the tenth iteration shows that PEKF-VB converge faster than CODEKF.

**Figure 8:**
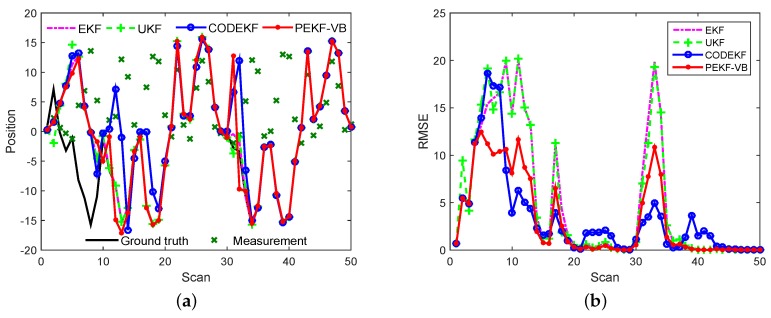
Comparison of EKF, UKF, CODEKF and PEKF-VB (**a**) Filtering results (**b**) RMSE.

Example 3: A Strongly nonlinear filtering problem. For further verifying our proposed method, we run EKF, UKF, CODEDK and PEKF-VB on the benchmark nonlinear problem in [32,33,34]

(40) $$\begin{array}{cc} x_{k}= & \frac{x_{k-1}}{2}+\frac{25x_{k-1}}{1+x_{k-1}^{2}}+8\cos\left(1.2k\right)+\nu_{k-1}, \end{array}$$

(41) $$\begin{array}{cc} z_{k}= & \frac{{x_{k}}^{2}}{20}+\omega_{k}, \end{array}$$

where $\nu_{k-1}$ and $\omega_{k}$ are zero mean Gaussian noise with variances $Q_{k-1}$ and $R_{k}$, respectively. We let $Q_{k}=0.0001$, $R_{k}=1$ and scan period T=1. The simulation results are given in Figure 8, from which we can see that CODEKF and PEKF-VB have very similar results and outperform EKF and UKF. This is because that local linearization adopted by EKF-based filter are not a sufficient description of the nonlinear nature of this example [34], while the VB iteration can make use of measurement information as much as possible.

## 6. Conclusions

We have developed a proximal iterative nonlinear filter, in which the expectation of the posterior PDF is approximated by a parameterised variational distribution that is iteratively optimized in the VB framework. A weighted KL divergence is adopted as a proximal term in the iteration to ensure the convergence can be achieved with a tight bound. The simulation results show that the proposed algorithm is better than several existing algorithms in terms of estimation accuracy at the cost of increased computational burden.

## Figures and Tables

**Table 1:** The quantitative comparison of the mean RMSE (from 20 to 80 scan) and the running time.

Algorithm	EKF	UKF	CODEKF	PEKF-VB
**Time ratio**	1	3.64	3.96	6.76
**Mean RMSE**	17.8129	17.8455	16.7758	16.3958

## Appendix A. Derivations of $\alpha_{k}^{i}$ and $\gamma_{k}^{i}$

Since the system is Gaussian, the likelihood function $p(z_{k}|x_{k|k}^{i})$ is

(A1) $$\begin{array}{cc} p(z_{k}|x_{k|k}^{i}) & =\frac{1}{\sqrt{{\left(2\pi \right)}^{1/D_{z}}\left|R_{k}\right|}}\exp\left(-\frac{1}{2}{\left(z_{k}-h_{k}\left(x_{k|k}^{i}\right)\right)}^{T}R_{k}^{-1}\left(z_{k}-h_{k}\left(x_{k|k}^{i}\right)\right)\right). \end{array}$$

The parameters $\alpha_{k}$ in Equation (18) and $\gamma_{k}^{i}$ in Equation (19) are derived according to Equation (A2) and Equation (A3), respectively.

(A2) $$\begin{array}{cc} \alpha_{k}^{i} & =-\nabla_{x}E_{q}\left[\log p\left(z_{k}|x_{k|k}^{i}\right)\right]\\ & =-\nabla_{x}E_{q}\left[\log\left(\frac{1}{\sqrt{{\left(2\pi \right)}^{1/D_{z}}\left|R_{k}\right|}}\exp\left(-\frac{1}{2}{\left(z_{k}-h\left(x_{k|k}^{i}\right)\right)}^{T}R_{k}^{-1}\left(z_{k}-h\left(x_{k|k}^{i}\right)\right)\right)\right)\right]. \end{array}$$

(A3) $$\begin{array}{cc} \gamma_{k}^{i} & =-\nabla_{P}E_{q}\left[\log p\left(z_{k}|x_{k|k}^{i}\right)\right]\\ & =-\nabla_{P}E_{q}\left[\log\left(\frac{1}{\sqrt{{\left(2\pi \right)}^{1/D_{z}}\left|R_{k}\right|}}\exp\left(-\frac{1}{2}{\left(z_{k}-h\left(x_{k|k}^{i}\right)\right)}^{T}R_{k}^{-1}\left(z_{k}-h\left(x_{k|k}^{i}\right)\right)\right)\right)\right]. \end{array}$$

By Bonnet’s theorem [35], the gradient of the expectation of $f\left(\xi \right)$ under a Gaussian distribution $N\left(\xi |\mu ,C\right)$ w.r.t. the mean μ is the expectation of the gradient of $f\left(\xi \right)$

(A4) $$\nabla_{\mu }E_{N\left(\xi |\mu ,C\right)}\left[f\left(\xi \right)\right]=E_{N\left(\xi |\mu ,C\right)}\left[\nabla_{\xi }f\left(\xi \right)\right].$$

It follows that $\alpha_{k}^{i}$ can be written

(A5) $$\begin{array}{cc} \alpha_{k}^{i} & =\frac{1}{2}E_{q}\left[\nabla_{x}\left({\left(z_{k}-h\left(x_{k|k}^{i}\right)\right)}^{T}R_{k}^{-1}\left(z_{k}-h\left(x_{k|k}^{i}\right)\right)\right)\right]\\ & =-{\left(H_{k}^{i}\right)}^{T}R_{k}^{-1}\left(z_{k}-h\left(x_{k|k}^{i}\right)\right), \end{array}$$

where the Jacobian matrix $H_{k}^{i}=\frac{\partial h_{k}\left(x\right)}{\partial x^{T}}{|}_{x=x_{k|k}^{i}}$.

According to Price’s theorem [36], the gradient of the expectation of $f\left(\xi \right)$ under a Gaussian distribution $N\left(\xi |\mu ,C\right)$ w.r.t. the covariance *C* is the expectation of the Hessian of $f\left(\xi \right)$:

(A6) $$\nabla_{c}E_{N\left(\xi |\mu ,C\right)}\left[f\left(\xi \right)\right]=\frac{1}{2}E_{N\left(\xi |\mu ,C\right)}\left[\nabla_{\xi ,\;\xi }f\left(\xi \right)\right].$$

Similarly,

(A7) $$\begin{array}{cc} \gamma_{k}^{i} & =-\frac{1}{2}E_{q}\left[\nabla_{x,x}\left({\left(z_{k}-h\left(x_{k|k}^{i}\right)\right)}^{T}R_{k}^{-1}\left(z_{k}-h\left(x_{k|k}^{i}\right)\right)\right)\right]\\ & =-\frac{1}{2}E_{q}\left[\nabla_{x}\left({\left(H_{k}^{i}\right)}^{T}\left({\left(R_{k}^{-1}\right)}^{T}+R_{k}^{-1}\right)\left(z_{k}-h\left(x_{k|k}^{i}\right)\right)\right)\right]\\ & =\frac{1}{2}{\left(H_{k}^{i}\right)}^{T}R_{k}^{-1}H_{k}^{i}. \end{array}$$

## Appendix B. Derivation of ELBO

From Equations (9) and (8), the ELBO is

(A8) $$L\left(\psi_{k}\right)=E_{q}\left[\log p\left(z_{k}|x_{k}\right)\right]+E_{q}\left[\log p\left(x_{k}\right)\right]-E_{q}\left[\log q\left(x_{k}|\psi_{k}\right)\right].$$

Since both system noise and measurement noise are Gaussian, the likelihood function $p\left(y_{k}|x_{k}\right)$, the PDF $p\left(x_{k}\right)$ and the PDF $q\left(x_{k}|\psi_{k}\right)$ below are Gaussian

(A9) $$\begin{array}{cc} p(z_{k}|x_{k})= & \frac{1}{\sqrt{{\left(2\pi \right)}^{D_{z}}|R_{k}}|}\exp\left(-\frac{1}{2}{\left(z_{k}-h\left(x_{k}\right)\right)}^{T}{R_{k}}^{-1}\left(z_{k}-h\left(x_{k}\right)\right)\right), \end{array}$$

(A10) $$\begin{array}{c} p\left(x_{k}\right)=\frac{1}{\sqrt{{\left(2\pi \right)}^{D_{x}}|Q_{k}}|}\exp\left(-\frac{1}{2}{\left(x_{k}-f_{k}\left(x_{k-1}\right)\right)}^{T}Q_{k}^{-1}\left(x_{k}-f_{k}\left(x_{k-1}\right)\right)\right), \end{array}$$

(A11) $$\begin{array}{cc} q\left(x_{k}|\psi_{k}\right) & =\frac{1}{\sqrt{{\left(2\pi \right)}^{D_{x}}|P_{k|k}}|}\exp\left(-\frac{1}{2}{\left(x_{k}-x_{k|k}\right)}^{T}P_{k|k}^{-1}\left(x_{k}-x_{k|k}\right)\right). \end{array}$$

Assume that the state transition matrix and the measurement matrix are obtained by linearization, $p(z_{k}|x_{k})$ and $p(x_{k})$ can be written as,

(A12) $$\begin{array}{cc} p(z_{k}|x_{k})\approx & \frac{1}{\sqrt{{\left(2\pi \right)}^{D_{z}}|R_{k}}|}\exp\left(-\frac{1}{2}{\left(z_{k}-H_{k}x_{k}\right)}^{T}{R_{k}}^{-1}\left(z_{k}-H_{k}x_{k}\right)\right), \end{array}$$

(A13) $$\begin{array}{c} p\left(x_{k}\right)\approx \frac{1}{\sqrt{{\left(2\pi \right)}^{D_{x}}|Q_{k}}|}\exp\left(-\frac{1}{2}{\left(x_{k}-F_{k-1}x_{k-1}\right)}^{T}Q_{k}^{-1}\left(x_{k}-F_{k-1}x_{k-1}\right)\right). \end{array}$$

where $F_{k-1}=\frac{\partial f\left(x\right)}{\partial x}{|}_{x=x_{k-1|k-1}}$ and $H_{k}=\frac{\partial h\left(x\right)}{\partial x}{|}_{x=x_{k|k}}$.

Assume that the state estimation is unbiased; that is, $E_{q}\left[x_{k-1}\right]=x_{k-1|k-1}$, Equation (A10) can be written approximately as

(A14) $$\begin{array}{cc} p\left(x_{k}\right) & =\frac{1}{\sqrt{{\left(2\pi \right)}^{D_{x}}|F_{k-1}P_{k-1|k-1}F_{k-1}^{T}+Q_{k-1}|}}\\ & \times \exp\left(-\frac{1}{2}{\left(x_{k}-F_{k-1}x_{k-1|k-1}\right)}^{T}{\left(F_{k-1}P_{k-1|k-1}F_{k-1}^{T}+Q_{k-1}\right)}^{-1}\left(x_{k}-F_{k-1}x_{k-1|k-1}\right)\right)\\ & =\frac{1}{\sqrt{{\left(2\pi \right)}^{D_{x}}|P_{k|k-1}}|}\exp\left(-\frac{1}{2}{\left(x_{k}-x_{k|k-1}\right)}^{T}P_{k|k-1}^{-1}\left(x_{k}-x_{k|k-1}\right)\right). \end{array}$$

Since estimation is unbiased, the ground truth $x_{k}$ at time *k* can be expressed as,

(A15) $$\begin{array}{c} x_{k}=x_{k|k}+{\tilde{x}}_{k|k}, \end{array}$$

where $E_{q}\left[{\tilde{x}}_{k|k}\right]=0$. The first term $E_{q}\left[\log p\left(z_{k}|x_{k}\right)\right]$ in Equation (A8) becomes

(A16) $$\begin{array}{cc} E_{q}\left[\log p\left(z_{k}|x_{k}\right)\right]\approx & -\frac{1}{2}\log\left({\left(2\pi \right)}^{D_{z}}\left|R_{k}\right|\right)\\ & -\frac{1}{2}\mathrm{tr}\left(R_{k}^{-1}E_{q}\left[\left(z_{k}-H_{k}\left(x_{k|k}+{\tilde{x}}_{k|k}\right)\right){\left(z_{k}-H_{k}\left(x_{k|k}+{\tilde{x}}_{k|k}\right)\right)}^{T}\right]\right)\\ = & -\frac{1}{2}\log\left({\left(2\pi \right)}^{D_{z}}\left|R_{k}\right|\right)\\ & -\frac{1}{2}\mathrm{tr}\left(R_{k}^{-1}\left(\left(z_{k}-H_{k}x_{k|k}\right){\left(z_{k}-H_{k}x_{k|k}\right)}^{T}+H_{k}P_{k|k}H_{k}^{T}\right)\right). \end{array}$$

The second term $E_{q}\left[\log p(x_{k})\right]$ in Equation (A8) becomes

(A17) $$\begin{array}{cc} E_{q}\left[\log p(x_{k})\right]\approx & -\frac{1}{2}\log\left({\left(2\pi \right)}^{D_{x}}\left|P_{k|k-1}\right|\right)-\frac{1}{2}\mathrm{tr}\left(P_{k|k-1}^{-1}E_{q}\left[\left(x_{k}-x_{k|k-1}\right){\left(x_{k}-x_{k|k-1}\right)}^{T}\right]\right)\\ = & -\frac{1}{2}\log\left({\left(2\pi \right)}^{D_{x}}\left|P_{k|k-1}\right|\right)-\frac{1}{2}\mathrm{tr}\left(P_{k|k-1}^{-1}E_{q}\left[\left(x_{k|k}+{\tilde{x}}_{k}\right){\left(x_{k|k}+{\tilde{x}}_{k}\right)}^{T}\right.\right.\\ & \left.\left.-\left(x_{k|k}+{\tilde{x}}_{k}\right)x_{k|k-1}^{T}-x_{k|k-1}{\left(x_{k|k}+{\tilde{x}}_{k}\right)}^{T}+x_{k|k-1}x_{k|k-1}^{T}\right]\right)\\ = & -\frac{1}{2}\log\left({\left(2\pi \right)}^{D_{x}}\left|P_{k|k-1}\right|\right)-\frac{1}{2}\mathrm{tr}\left(P_{k|k-1}^{-1}P_{k|k}\right)\\ & -\frac{1}{2}\mathrm{tr}\left(P_{k|k-1}^{-1}E_{q}\left[x_{k|k}x_{k|k}^{T}-x_{k|k}x_{k|k-1}^{T}-x_{k|k-1}x_{k|k}^{T}+x_{k|k-1}x_{k|k-1}^{T}\right]\right)\\ = & -\frac{1}{2}\log\left({\left(2\pi \right)}^{D_{x}}\left|P_{k|k-1}\right|\right)-\frac{1}{2}\mathrm{tr}\left(P_{k|k-1}^{-1}P_{k|k}\right)\\ & -\frac{1}{2}\mathrm{tr}\left(P_{k|k-1}^{-1}\left(\left(x_{k|k}-x_{k|k-1}\right){\left(x_{k|k}-x_{k|k-1}\right)}^{T}\right)\right). \end{array}$$

The third term $E_{q}\left[\log q\left(x_{k}|\psi_{k}\right)\right]$ in Equation (A8) is

(A18) $$\begin{array}{cc} E_{q}\left[\log q\left(x_{k}|\psi_{k}\right)\right] & \approx -\frac{1}{2}\log\left({\left(2\pi \right)}^{D_{x}}\left|P_{k|k}\right|\right)-\frac{1}{2}\mathrm{tr}\left(P_{k|k}^{-1}E_{q}\left[\left(x_{k}-x_{k|k}\right){\left(x_{k}-x_{k|k}\right)}^{T}\right]\right)\\ & =-\frac{1}{2}\log\left({\left(2\pi \right)}^{D_{x}}\left|P_{k|k}\right|\right)-\frac{D_{x}}{2}, \end{array}$$

Combining Equations (A16)–(A18), we obtain the ELBO as

(A19) $$\begin{array}{cc} L\left(\psi_{k}\right)= & -\frac{1}{2}\log\frac{{\left(2\pi \right)}^{D_{z}}|R_{k}\left|\right|P_{k|k-1}|}{|P_{k|k}|}-\frac{1}{2}\mathrm{tr}\left(P_{k|k-1}^{-1}\left[\left(x_{k|k}-x_{k|k-1}\right){\left(x_{k|k}-x_{k|k-1}\right)}^{T}\right]\right)\\ & -\frac{1}{2}\mathrm{tr}\left(P_{k|k-1}^{-1}P_{k|k}\right)-\frac{1}{2}\mathrm{tr}\left(R_{k}^{-1}\left(\left(z_{k}-H_{k}x_{k|k}\right){\left(z_{k}-H_{k}x_{k|k}\right)}^{T}+H_{k}P_{k|k}H_{k}^{T}\right)\right)+\frac{D_{x}}{2}. \end{array}$$
